# The challenge of ventilator-associated pneumonia diagnosis in COVID-19 patients

**DOI:** 10.1186/s13054-020-03013-2

**Published:** 2020-06-05

**Authors:** Bruno François, Pierre-François Laterre, Charles-Edouard Luyt, Jean Chastre

**Affiliations:** 1 Intensive Care Unit and Inserm CIC1435 & UMR1092, CHU Dupuytren, Limoges, France; 2 Intensive Care Unit, St Luc University Hospital, Université Catholique de Louvain, Brussels, Belgium; 3 Intensive Care Unit, Hôpital La Pitié-Salpétrière, APHP-Sorbonne Université, Paris, France

While different phenotypes have been evidenced in ICU COVID-19 pneumonia [1], most patients meet ARDS Berlin definition associating bilateral radiologic infiltrates with severe hypoxemia. COVID-19 patients frequently require prolonged invasive mechanical ventilation (MV) including prone positioning, heavy sedation, and muscle blockers for several weeks. In addition, there is clear evidence of prolonged immunosuppression including deep lymphopenia [2]. This accounts for a high risk of secondary hospital-acquired infections, primarily ventilator-associated pneumonia (VAP). The diagnosis of ventilator-associated infections remains challenging due to major definition heterogeneity of multiple clinical entities, and no consensus has yet been reached on appropriate diagnostic strategies for VAP. Irrespective of the definition, accurate diagnosis of VAP requires clinical signs of infection, microbiological documentation, and chest X-ray findings, even if the latter may be difficult to interpret due to preexisting parenchymal injury [3].

The clinical presentation of COVID-19 pneumonia is relatively homogenous and commonly associates high fever, hyperleukocytosis, severe hypoxemia, extensive bilateral radiologic infiltrates, and biological inflammatory syndrome. Since this presentation is shared with VAP, traditional diagnostic criteria for VAP are not valid in the critical COVID-19 population. Similarly, the Clinical Pulmonary Infection Score (CPIS), assessing body temperature, tracheal secretions, radiologic infiltrate, hyperleukocytosis, and PaO_2_/FiO_2_, has little additional value since its components overlap with those of COVID-19 pneumonia in MV patients [4]. In our experience, more than 90% of COVID-19 patients had qualifying CPIS > 6 on day 2 following ICU admission in the absence of any documented VAP or co-infection. Accordingly, the microbiological documentation from deep respiratory secretions currently remains the sole criterion to support VAP diagnosis in COVID-19 patients. Fiberoptic broncho-alveolar lavage is hardly feasible in severely hypoxemic COVID-19 patients due to the inherent risk of worsening hypoxemia. Hence, many ICU perform less invasive endotracheal aspirate (ETA) with quantitative or semi-quantitative cultures, even if less reliable for deciding whether to institute antibiotic treatment or not. It is near impossible to distinguish COVID-19-associated ARDS with asymptomatic bacterial colonization from a true VAP based solely on traditional threshold values (i.e., 10^5^ CFU/ml for ETA). Interestingly, despite heavy bacterial load, white blood cell count in bronchial secretions appears very low in most COVID-19 patients developing a superinfection. Accurate identification of COVID-19 patients who require treatment with new antibiotics for a clinically relevant bacterial superinfection is difficult, leading to overuse of broad-spectrum antibiotics despite the absence of supporting data in the literature [5]. As a result, most ventilated COVID-19 patients with ARDS are treated with prophylactic antibiotics to prevent from undocumented VAP. Such a strategy is at high risk of selection of multi-drug-resistant bacteria or even fungi in patients expected to remain under invasive MV for a long period. The COVID-19 pandemic and the severity of its clinical presentation cannot justify “emotional” and blind antibiotic therapy on the sole argument that traditional VAP definition is invalid. Specific COVID-19 antimicrobial stewardship and guidelines are required to avoid this detrimental approach, considering that within the first 10 days, most of the pathogens documented in the lung are from the community with minimal resistant profile (unpublished data). While it is reasonable to initiate antibiotics in patients with suspected VAP and hemodynamic instability or severe hypoxemia following European guidelines [6] regardless of clinical certainty, a more conservative approach may be beneficial for stable patients (Fig. 1). It remains to be seen whether routine assessment tools such as daily variations of CPIS score, serial viral load aspirates, new molecular techniques, or lung ultrasonography will help improving decisions regarding antibiotic treatment in such a clinically complex population. Diagnostic algorithms using a PCT-guided strategy for stopping early empiric antimicrobial treatment [7] or pathogen quantification trends could be alternatively tested for VAP diagnosis. Overall, studies in this field are urgently needed.

**Fig. 1:**
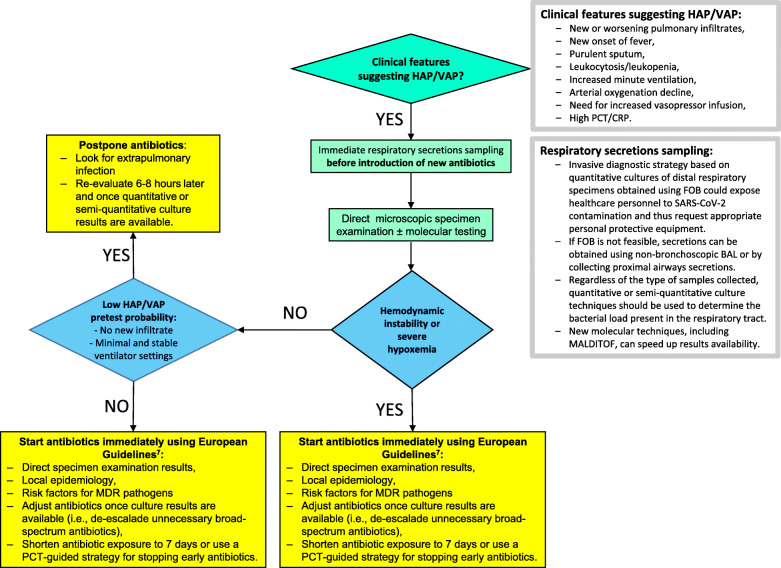
Empiric antibiotic treatment decision tree for HAP/VAP in COVID-19 patients

## Data Availability

NA

## Abbreviations

ARDS: Acute respiratory distress syndrome
CPIS: Clinical Pulmonary Infection Score
ETA: Endotracheal aspirate
ICU: Intensive care unit
MV: Mechanical ventilation
PCT: Procalcitonin
VAP: Ventilator-associated pneumonia
FOB: Fiberoptic bronchoscopy
